# Prediction of dengue outbreaks based on disease surveillance, meteorological and socio-economic data

**DOI:** 10.1186/s12879-019-3874-x

**Published:** 2019-03-21

**Authors:** Raghvendra Jain, Sra Sontisirikit, Sopon Iamsirithaworn, Helmut Prendinger

**Affiliations:** 1 National Institute of Informatics, Tokyo, Japan; 2 Asian Institute of Technology, School of Engineering and Technology, Bangkok, Thailand; 3 Department of Disease Control Thirteenth Division, Bangkok, Thailand

**Keywords:** Dengue forecasting, Data-driven epidemiology, Disease surveillance, Generalized additive models (GAMs)

## Abstract

**Background:**

The goal of this research is to create a system that can use the available relevant information about the factors responsible for the spread of dengue and; use it to predict the occurrence of dengue within a geographical region, so that public health experts can prepare for, manage and control the epidemic. Our study presents new geospatial insights into our understanding and management of health, disease and health-care systems.

**Methods:**

We present a machine learning-based methodology capable of providing forecast estimates of dengue prediction in each of the fifty districts of Thailand by leveraging data from multiple data sources. Using a set of prediction variables, we show an increase in prediction accuracy of the model with an optimal combination of predictors which include: meteorological data, clinical data, lag variables of disease surveillance, socioeconomic data and the data encoding spatial dependence on dengue transmission. We use Generalized Additive Models (GAMs) to fit the relationships between the predictors (with a lag of one month) and the clinical data of Dengue hemorrhagic fever (DHF) using the data from 2008 to 2012. Using the data from 2013 to 2015 and a comparative set of prediction models, we evaluate the predictive ability of the fitted models according to RMSE and SRMSE as well as using adjusted R-squared value, deviance explained and change in AIC.

**Results:**

The model allows for combining different predictors to make forecasts with a lead time of one month and also describe the statistical significance of the variables used to characterize the forecast. The discriminating ability of the final model was evaluated against Bangkok specific *constant* threshold and WHO *moving* threshold of the epidemic in terms of specificity, sensitivity, positive predictive value (PPV), and negative predictive value (NPV).

**Conclusions:**

The out-of-sample validation showed poorer results than the in-sample validation, however it demonstrated ability in detecting outbreaks up-to one month ahead. We also determine that for the predicting dengue outbreaks within a district, the influence of dengue incidences and socioeconomic data from the surrounding districts is statistically significant. This validates the influence of movement patterns of people and spatial heterogeneity of human activities on the spread of the epidemic.

## Background

Dengue, a mosquito-borne viral disease, is caused by four distinct, but closely related, serotypes of the virus [1, 2]. Recovery from infection by one of these four (DEN-1, DEN-2, DEN-3, and DEN-4) provides the infected person lifelong immunity against that particular serotype and cross-immunity to the other serotypes. The time duration for this cross-immunity is 6-12 months [1]. If the person is infected by other serotypes subsequently then the risk of severe dengue increases. The uninfected mosquitoes get the virus from infected humans and thus the later becomes the primary carrier, multiplier, and transmitter of the DENV (dengue virus).

**Fig. 1:**
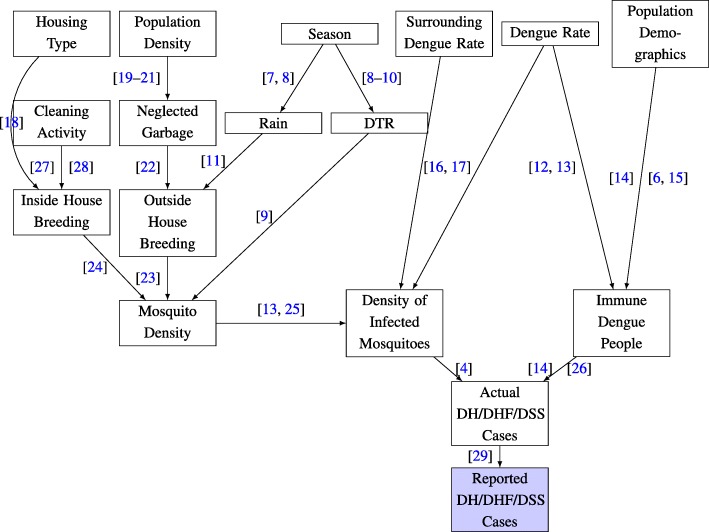
Dependency chart for the occurence of DH/DHF/DSS Cases

Thailand began to experience Dengue fever in 1949 and it became pandemic in the country for the first time in 1958 in Bangkok [3]. The information about the clinically diagnosed cases of dengue fever(DF), dengue hemorrhagic fever (DHF) and dengue shock syndrome (DSS) are sent to the Epidemiology Department in Bangkok [4] via the Provincial Health Offices. The transmission of DENV which occurs through the bite of infected Aedes mosquitoes, principally Aedes aegypti, has dramatically increased in the last two decades [5] and occurrence of dengue fever is likely to rise [6] due to the combination of several direct and indirect factors. The causal dependency of some of the prominent factors on DENV transmission is shown shown in Fig. 1.

These factors include density of infected mosquitoes, (level of) immunity of people to dengue serotypes, meteorology, human related factors e.g. housing type, population density and demographics, cleanliness etc. Several potential predictive indicators that contribute to the above mentioned factors have been described [23, 30–32]. For example, the density of infected mosquitoes depends on total mosquito density [13, 25] which depends on inside house breeding [24] and outside house breeding [23]. The inside house breeding depends on cleaning activity [27, 28] and housing type [18] among other factors whereas outside house breeding is primarily influenced by neglected garbage [22], population density [19–21] and rain [11]. The seasonal influence on rain [7, 8] and temperature [8–10] contributes to the mosquito density [9, 11, 23, 32, 33]. The density of infected mosquitoes in the region is influenced by dengue rate in the region [16, 17] and so is the number of people with immunity [12, 13] along with the population demographics [6, 14, 15].

Despite the numerous urban outbreaks of dengue with significant health and economic impact [34–37], the detailed surveillance for diagnosing dengue has been limited which makes it difficult to generate detailed information on its epidemiology [38, 39]. Moreover, there are currently no licensed vaccines or specific therapeutics for the treatment of the infected people. Thus, effective vector control interventions are the only way to control the transmission of dengue and other Aedes-borne arboviral diseases. A variety of dengue vector control strategies [6] have been adopted in different regions [40–43] but this has not stopped its rapid emergence and global spread [44].

The complexity of early warning systems (EWS) arise due to the involvement of various factors such as environmental, climatic or geographic ones along with the well-studied transmission patterns between the different animal, human or vector components. Prediction forms an important part of surveillance systems and more specifically in EWS. To predict the future outbreaks using information on the risk factors of the disease, epidemiological models have been proposed ([45] for a review). These prediction models help in decision making processes concerning control purposes and surveillance methods. A large number of them [46–48] focused on modeling climate impact (temperature and precipitation data) on dengue transmission. In a few studies, along with the climate data, other covariates were also used on a longitude-latitude grid with time lags relevant to dengue transmission. These covariates incorporated relevant socio-economic and environmental variables [49, 50], socio-geographical factors [51, 52], imported cases [53, 54] as well human movement patterns [55, 56]. We found that for the modeling process, many previous studies have not sufficiently accounted for the *integration* of spatio-temporal features of the disease, its socio-environmental aspects and factors due to increased movements of people in a single prediction model. However, incorporating such information and understanding the relative importance of one risk factor over the other is important for their use in an early warning system.

Therefore, the goal of this research is to create a system that can use the available relevant information about the factors responsible for the spread of dengue and; use it to predict the occurrence of dengue within a geographical region, so that public health experts can prepare for, manage and control the epidemic. Our study presents new geospatial insights into our understanding and management of health, disease and health care systems. It yields practical results (e.g., results of value to a national public health, control, screening or prevention program, or local resource planning program along with serving to re-demonstrate a previously well-documented phenomenon.

## Methods

### Study area

**Fig. 2:**
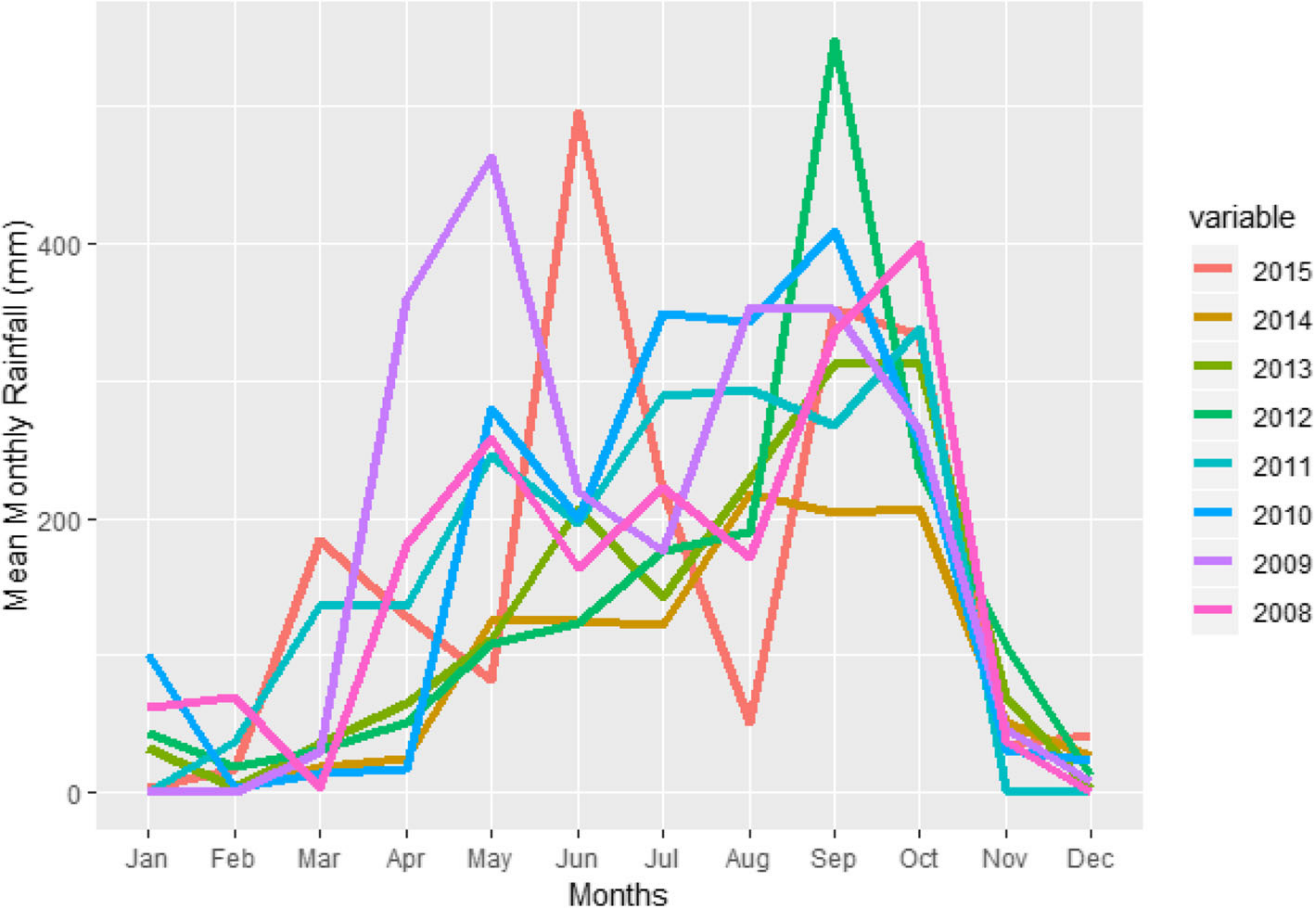
Mean Monthly Rainfall within Bangkok for different the years

**Fig. 3:**
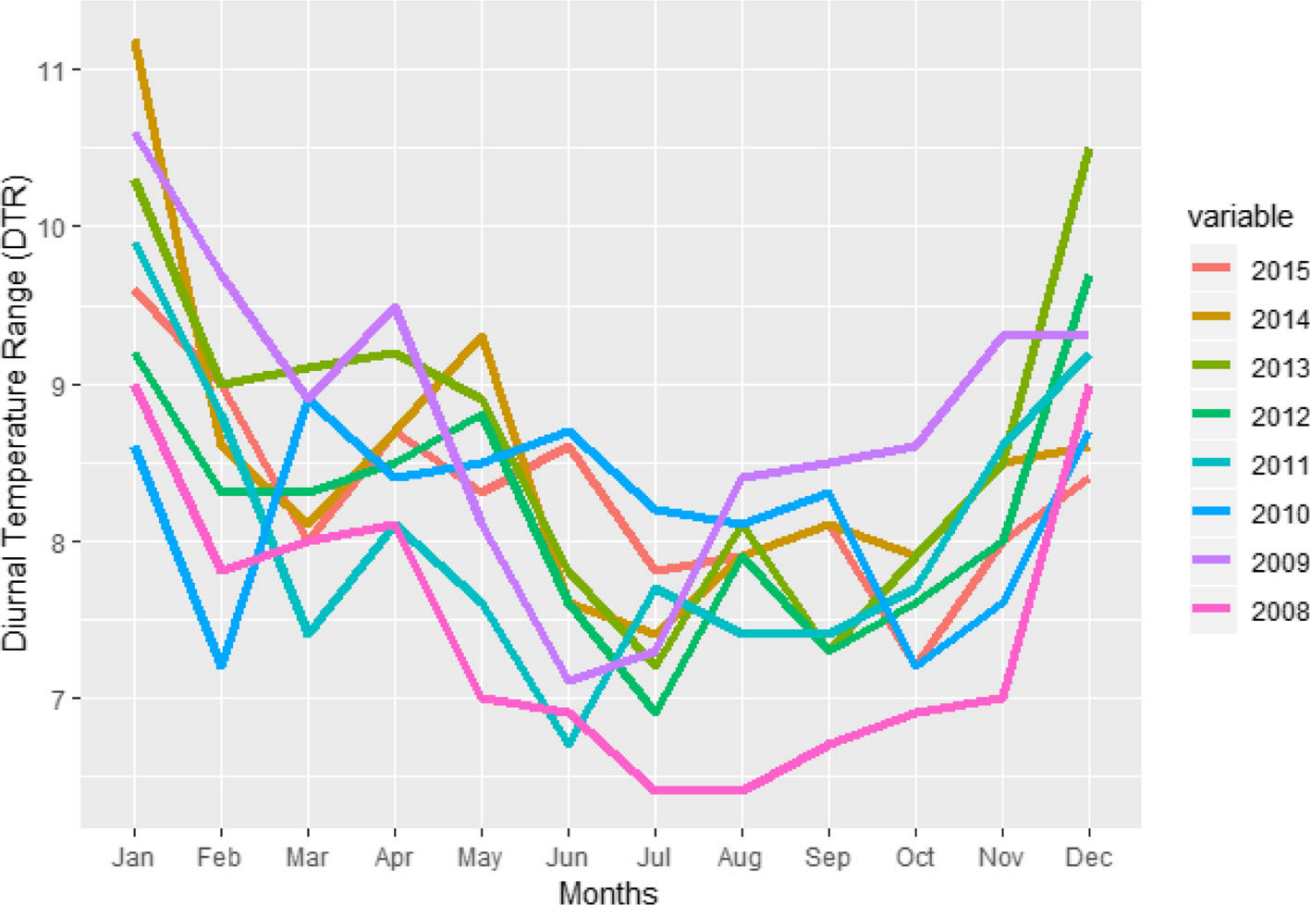
Monthly DTR within Bangkok for different years

The dengue outbreak in Bangkok can influence the dengue situation for the whole country because Bangkok is a very crowded city located at the center of Thailand. In the 2010 census, the population of Bangkok was about 8.28 million, although only about 5.7 million were the registered residents. The population within the city during the day swells to about to 15 million [7] due to the commuters from the surrounding areas. During a winter season, the temperature in Bangkok is still high around 28-35 degree Celsius and there is rain in every season [57]. Figures 2 and 3 show the cumulative monthly rainfall and monthly diurnal temperature range (DTR) of Bangkok throughout the years (2008−2015). The diurnal temperature range (DTR) is the difference between the daily maximum and minimum temperature. The experimental evaluation has shown that DTR influences two important parameters underlying dengue virus (DENV) transmission by Aedes mosquito [9].

Moreover, many people who live in Bangkok travel to other provinces frequently, so these people may be the main carriers and multipliers of the virus that cause dengue outbreak in other parts of Thailand. The Bangkok city covers an area of 1,568.737 square kilometers and it is subdivided into 50 districts, which are further sub-divided into 169 sub-districts. The total population registered in Bangkok is 5,693,884 and more than three million unregistered people live in Bangkok. The average registered population of Bangkok districts in 2014 is 113845.

### Data

Climate is one of the main factor related to dengue outbreak both locally and globally [58, 59]. Most of Thailand has a tropical wet and dry climate type, making the city amenable for Aedes mosquito to breed and spread in any season. The role of the variation in climatic factors on transmission dynamics and the geographic distribution of dengue has been well-studied [60]. The rainy season allows Aedes mosquitoes egg to grow into adult mosquitoes easier than in dry season. The increase of Aedes mosquitoes is directly affecting dengue cases in Thailand as shown in Fig. 5.

**Fig. 4:**
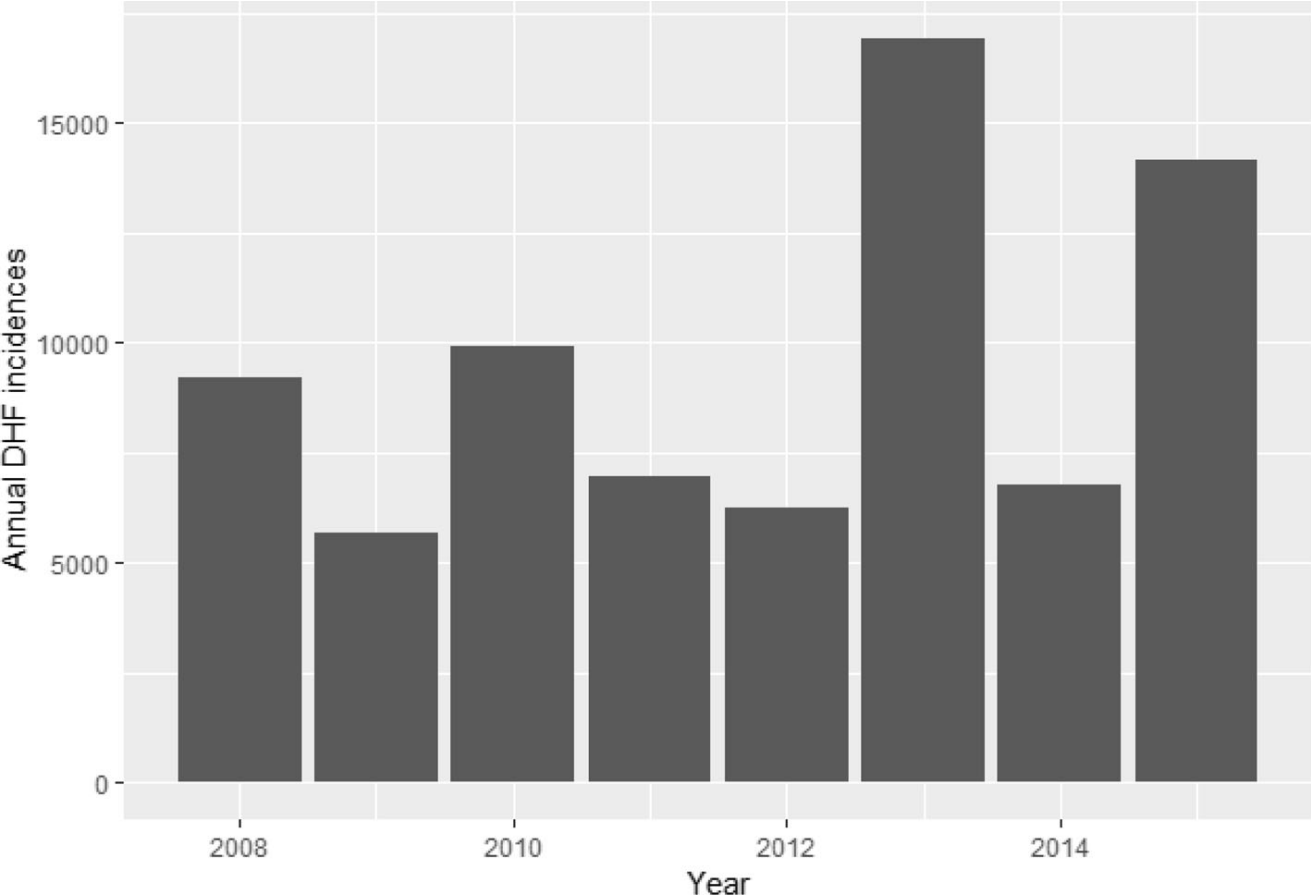
Data suggests that the outbreak increases every alternate year

**Fig. 5:**
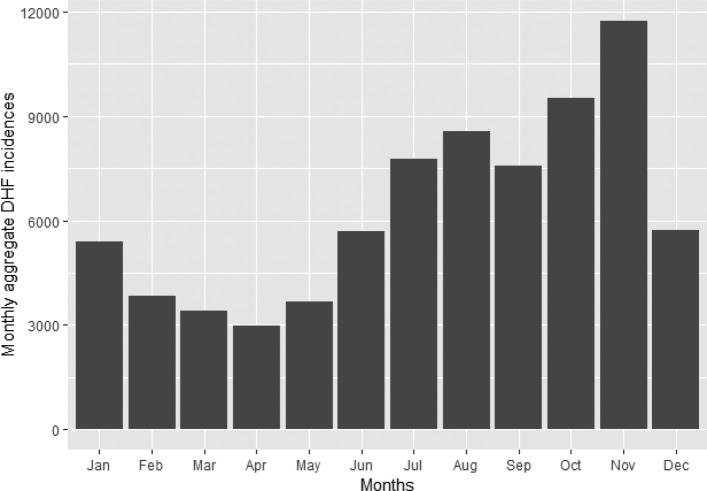
The DHF incidence peaked in October and November. Graph shows the amount of dengue patients for each month for the years 2008–2015. Rainy season continues from mid-May to mid-October

Figure 4 shows the increase of DHF cases in Bangkok up to 2015. The average number of DHF cases in Bangkok from 2008 until 2015 is around 4,750 cases per year. The highest number of DHF cases in Bangkok is 16942 which happened in 2013 followed by 14154 cases in 2015. As shown in Fig. 5, the highest number of monthly DHF cases in Bangkok is 11752 in the month of November followed by 9511 cases in October.

**Fig. 6:**
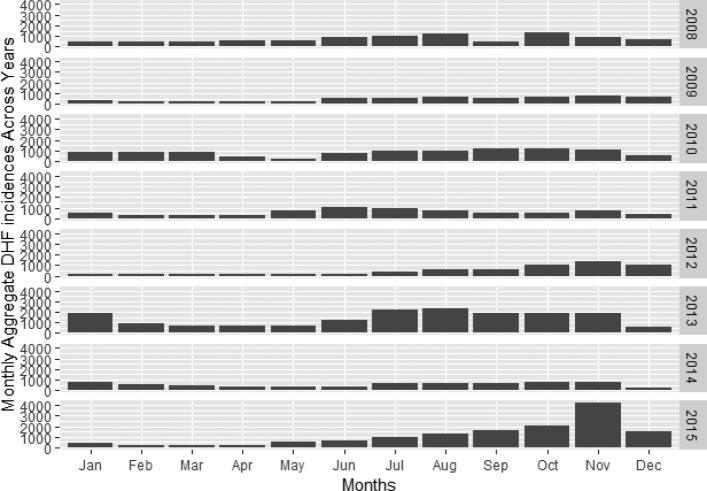
The DHF incidence peaks in the months of October and November. However in the year 2013, DHF outbreak happened for several months continuously

Figure 6 shows the variation of dengue cases with months. In the rainy season (mid-May–October), dengue cases rise dramatically, and then they decrease suddenly in summer season (February—mid-May). The reason that November has the highest number of cases is that dengue virus in patients need 4–10 days for the incubation period. So most dengue patients in November are infected in the rainy season (October-November).

In this study, we have used aggregated monthly dengue cases (dengue hemorrhagic fever) at district level from the Department of Disease Control, Bangkok for the period 2008−2015. Rainfall data from 2008−2015 is provided by the Department of Drainage and Sewerage, Bangkok and the temperature data for the same period is provided by the Meteorological Department, Thailand. Records of cumulative monthly rainfall (mm) and monthly DTR readings (°C) were obtained. The meteorological data were merged to the aggregated total number of confirmed dengue cases per month in each district of Bangkok. Using the geographical information of different districts of Bangkok, this data of each target district was merged to the aggregated total number of confirmed dengue cases per month in each of its surrounding district. Thus, our data has (a) Dengue virus prediction variables (Monthly DHF incidents in a district + aggregated monthly DHF incident in the nearby districts) and (b) Aedes mosquito prediction variables (cumulative monthly rainfall in the Bangkok + mean monthly average diurnal temperature range (DTR) in Bangkok).

A model was developed and validated by dividing the data file into two data sets: one for training the model and another for testing and validation of the fitted model. Here we varied the period in both the data sets. For example, in one case of prediction the data from January 2008 to December 2012 was used to train a model, and data from January 2013 to December 2015 was used for testing and validation of the fitted model. All the analyses were performed in R [62] using the mgcv package [63]. The details of the different such cases of predictions are listed in Table 2.

### Statistical analysis

These analyses focus on studying the following relationships:

- relationship of meteorological (DTR and rainfall) and socioeconomic data (monthly garbage collection in each district) to the time series of dengue incidences in that particular district.
- relationship of dengue transmission in a specific month in a district with the data of its past occurrences.
- relationship of dengue transmission in a specific month in a district with the data of past occurrences of dengue in its surrounding districts.

We created a set of prediction models encoding the above-mentioned relationships. Our geospatial/statistical method used in our work and the approach using Generalized Additive Models (GAM) to derive the insights are similar to the research study [64] conducted in Indonesia. However, along with evaluating different predictions models, we evaluate a different/unique hypothesis using a novel data-set; the feature-set used in the study is richer and the study area/country is different from the above-mentioned work.

The target of prediction was the cumulative dengue count in Bangkok (i.e. sum total of dengue incidences in all the 50 districts of the city) in a particular month of the year. According to the previous studies to determine the optimal lead time for dengue forecasts [65], there is evidence of increasing dengue cases in lag time of up to 4–20 weeks. Thus, similar to [64] we decided a priori, for meteorological variables, to use lag times with up to 4 months delay (i.e. 0–3 months) in the analysis. Since the dengue counts vary within and between the years, the count data is likely to be over-dispersed. Thus, rather than using the “standard” Poisson regression in which it is assumed that variance of count data is constant regardless of the expected value, we adopt a Quasi-Poisson regression in which the variance of count data (dengue counts) is assumed to be a linear function of the mean. To allow for over-dispersion a log-link function of dengue count data is used. To allow for non-linear response and exposure association between the predictors and the dengue incidences, cubic splines of 3 degrees of freedom was applied on the meteorological variables. The generalized additive model (GAM) can be expressed as:

1 $$  \begin{aligned} log (C_{0,t}) \sim \alpha + \sum_{l=0}^{3} ns(\rlap{T}c_{lt}, d =3) + \sum_{l=0}^{3} ns(R_{lt}, d =3) \end{aligned}  $$

where log() denotes the natural logarithm, *C* represents the total dengue count data, *t* denotes to time in months, T c represents DTR (°C), *R* denotes the mean monthly rainfall (mm) and l, denotes the lag variables, ns() denotes a natural cubic spline.

High correlation among predictor variables may give rise to singularity problems when fitting a statistical model. However, for GAMs, checking for collinearity is not sufficient. Since, we are now fitting smooth functions; it should be determined whether the smooth function of one variable can be produced using a combination of the smooths of the other terms in the model. This is called checking for *concurvity*. We performed the concurvity check for all the predictor variables. For more information on multicollinearity and concurvity in some nonlinear multivariate models including GAMs, see [66]. For understanding the effects of concurvity while using GAMs in the context of epidemiological research, see [67, 68].

**Disease surveillance data of each districts as predictor:** Since the current number of dengue incidences are influenced by the number of cases in the past, to determine this period of influence we have considered two approaches. The first approach focuses on determining the optimal lag term for short-term lagged dengue incidence data. The auto-regressive patterns in dengue time series data were studied by fitting a GAM in which data up to a delay of 4 months was used (similar to what we did with the meteorological data). This model was to fit to assess the influence of past dengue incidence on current count independent of meteorological factors. The regression model can be expressed as:

2 $$  \begin{aligned} log (C_{0,t}) \sim \alpha + \sum_{l=1}^{4} ns(C_{lt}, d =3) \end{aligned}  $$

Second approach focused on assessing the risk of retrospective transmission (1–48 months) on dengue incidences. Prior studies conducted in the region have shown that cross-immunity for dengue virus serotypes may significantly alter the dengue transmission over a period [12, 69]. We hypothesize that this might partly explain bi-annual cyclic epidemic pattern of dengue occurrence in Bangkok as shown in Fig. 4. Thus, we assess and estimate the risk of retrospective dengue transmission up to 1–30 months on current dengue transmission. To incorporate the effects delayed in time, the statistical model of DLNM was used to describe the additional time dimension of this exposure-relationship [70]. The GitHub repository of R implementation of DLNM used for our analyses is available at https://github.com/gasparrini/dlnm. The regression model can be expressed as:

3 $$ \begin{aligned}  log (C_{0,t}) \sim \alpha + \sum_{l=1}^{30} DLNM(C_{lt}, d = 4) \end{aligned}  $$

The output from both these approaches was then combined to determine the ‘optimal lag’ of disease surveillance.

**Disease surveillance data from surrounding districts as predictor:** There is much research which concludes that many dengue cases that occur in urban areas are due to the factors such as high population density, inadequate housing, and inappropriate human behavioral practices [19–21]. Surveillance of Aedes mosquito density is important for construction models of dengue transmission, in order to prioritize areas and seasons for vector control. The 80% of larvae or pupa in house are from Aedes mosquito. A recent study [23] has explored the dengue occurrence in a region in relation to its surrounding regions. The study is conducted in near real-time using object-based and spatial metric approaches. The geospatial analysis conducted on the data acquired using Google search and advanced land observation satellite images suggests that the occurrence and spread of dengue cases are positively correlated with densely populated areas which are *surrounded by dense vegetation*. This further suggests that the spatial heterogeneity of human activities influence the dengue epidemic. Thus, to determine the influence of spatial heterogeneity of human activities ongoing in nearby areas, we consider the data (both ‘short-term’ and ‘long-term’) from the dengue incidences of surrounding districts. The regression model can be expressed as:

4 $$  {\begin{aligned} log (C_{0,t}) \sim \alpha + \sum_{l=1}^{4} ns(S_{lt}, d =3) + \sum_{l=0}^{30} DLNM(S_{lt}, d = 4) \end{aligned}}  $$

where log() denotes the natural logarithm, *C* represents the total dengue count data, *S* represents the count data of dengue incidence occurred in the surrounding districts, *t* denotes to time in months, *l* denotes the lag variables and ns() denotes a natural cubic spline.

**Waste disposal data from each district as predictor:** Previous studies have shown the spatial correlation of socioeconomic data and urbanization with dengue incidences [71, 72]. Since the waste disposal and landfill dumps are the spatial infrastructures of any modern city, we used the data about monthly garbage collection from each district as an indicator for social capital.

Thus using the above-mentioned predictors, we evaluated the following models:

(A) Seasonal Naïve model (each forecast is set to be equal to the last observed value from the same season of the year (e.g., the same month of the previous year).
(B) Meteorology Optimal model (includes lag of 0−4 months for DTR and mean monthly rainfall)
(C) Optimal Lag Surveillance Model (includes lagged dengue count data for 1, 2 and 23^*rd*^ months)
(D) Optimal Met and Lag Surveillance Model (includes lagged meteorology data for 1−3 months and lagged dengue count data for 1, 2 and 23^*rd*^ months)
(E) Optimal Representation Model Combination of (D) with lagged dengue count data of surrounding districts for 1^*st*^ and 2^*nd*^ months
(F) Social-economic data Included Combination of (E) with garbage collection data of each district as the social capital

For complex models (B)-(F), a necessary time latency of at least 1 month lag was evaluated to allow up to 1 month lead time in controlling the disease at forecast of epidemics.

## Results

**Fig. 7:**
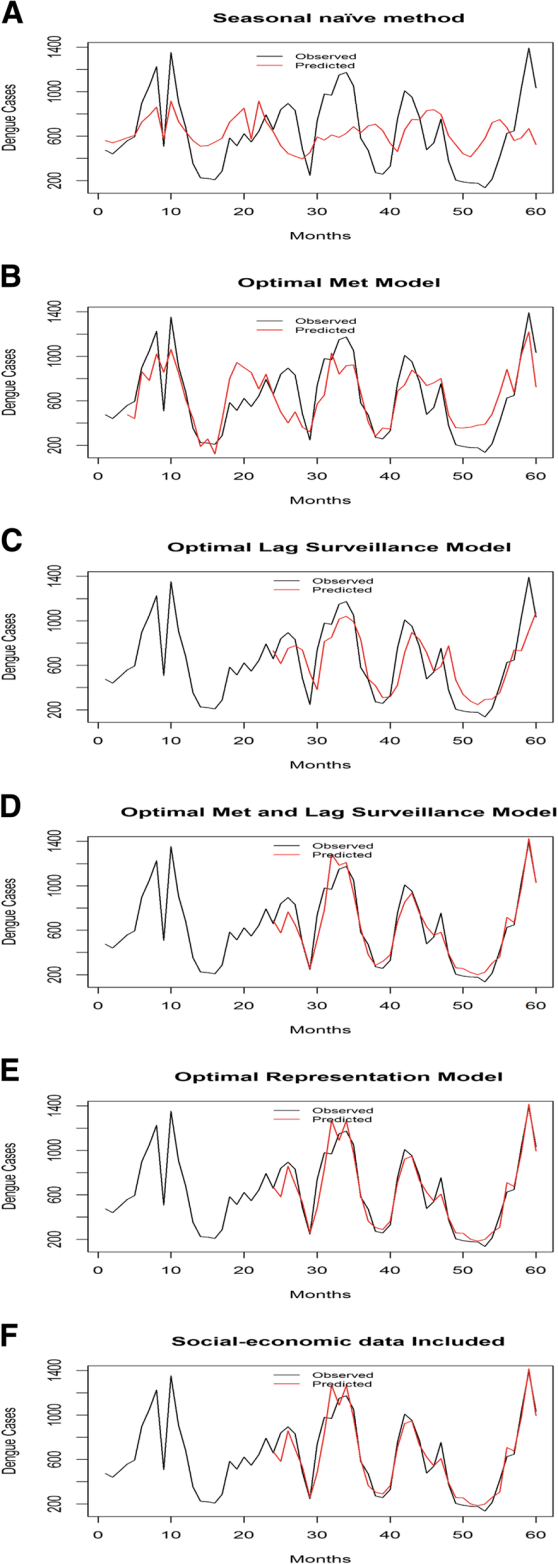
Monthly observed and predicted dengue cases from 2008-2012.

In this section, we explain about the models (A-F) listed in last section. The performance of these model is shown in Table 2 and the visual analyses of the prediction are shown in Fig. 7. The vertical axis in Fig. 7 represents the number of DHF cases and the horizontal axis depicts the time in months from 2008-2012. The errors RMSE/SRMSE are calculated by testing on in-sample data. The prediction performance is compared over the same time periods (months 23−60) to reduce the potential bias. This period is chosen because the final model uses lagged dengue data of past 23^*rd*^ month (as explained later in this section). The increase in adjusted R-squared value happens only if the new term improves the model more than what would be expected by chance (the higher the better). Deviance is a goodness-of-fit statistic for a statistical model (the higher the better) and the larger difference in AIC indicates stronger evidence for one model over the other (the lower the better). For comparing the difference in AIC, all the fitted models (B-F) are compared to base model A (Seasonal Naïve model). The discriminating ability of the the finally selected model was evaluated against Bangkok specific and WHO threshold of the epidemic in terms of specificity, sensitivity, positive predictive value (PPV), and negative predictive value (NPV).

As a first step for model fitting, the data for lags of month 0–3 of Diurnal Temperature Range (DTR) and monthly average rainfall was used to analyze the (1) statistical significance of the model terms (2) association of DTR and rainfall with dengue cases. Table 1 shown the approximate statistical significance of the smooth terms for DTR and rainfall.

**Table 1:** Approximate significance of smooth terms depicting lagged meteorological data

Results				
*P*-Value	Lag 0	Lag 1	Lag 2	Lag 3
Mean Monthly DTR	5.26e-16	0.000261	0.000921	2e-16
Cumulative Monthly Rainfall	8.96e-15	2e-16	2e-16	8.77e-10
R-sq(adj.)	0.283			
Deviance Explained	31.9%			
RMSE	8.462			
SRMSE	0.52

**Table 2:** Predictive performance statistics of different models evaluated on the training data for the same time period (months 23−60) to reduce the potential bias

Model Name	RMSE	SRMSE	R-sq.(adj)	Deviance Explained	*Δ* AIC
A: Seasonal Naïve	10.22	0.62	0.16	0.16	0
B: Meteorology Optimal	8.83	0.54	0.28	0.32	-492.64
C: Optimal Lag Surveillance Model	7.32	0.45	0.49	0.49	-2420.39
D: Optimal Met and Lag Surveillance Model	6.30	0.39	0.62	0.64	-2725.62
E: Optimal Representation Model	6.12	0.37	0.64	0.66	-2718.90
F: Social-economic data Included	6.10	0.37	0.64	0.73	-2713.86

The performance is measured on different metrics. The best model should have the lowest errors (RMSE, SRMSE) and have the best fit (measured in R-sq.(adj).), high deviance and low *Δ* AIC

**Fig. 8:**
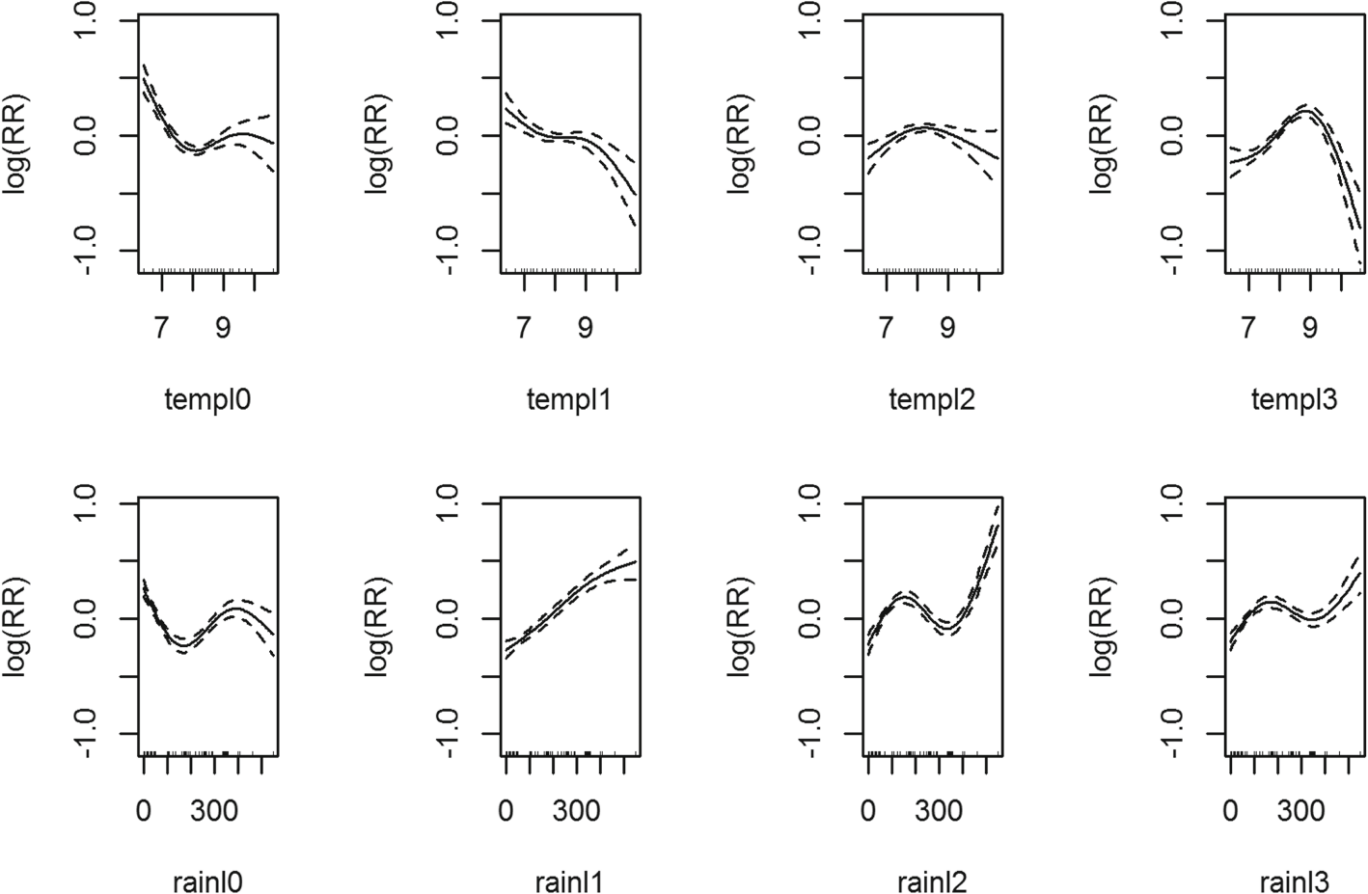
Association between the meteorological variables and dengue over lags of 0–3 months. Solid lines represent relative risks (RR) of dengue cases whereas the dotted lines depict the upper and lower limits of 95% confidence intervals. *P*-values and the initial analyses are listed in Table 1

As shown in Fig. 8, the association of temperatures with dengue cases in lag 0,1 decreases when the DTR is less than 8 °C and then increases. However, for the lag 3, this association shows a slight increase and then decrease. For lag 4, it is observed that the association of DTR with dengue cases increases linearly when the temperature is less than 9 °C and then a strong drop is observed. Due to the statistical significance of these terms i.e. lags of month 0–3 for the meteorological data, we call the model comprising of them as ‘Optimal Met’ model (model B).

**Fig. 9:**
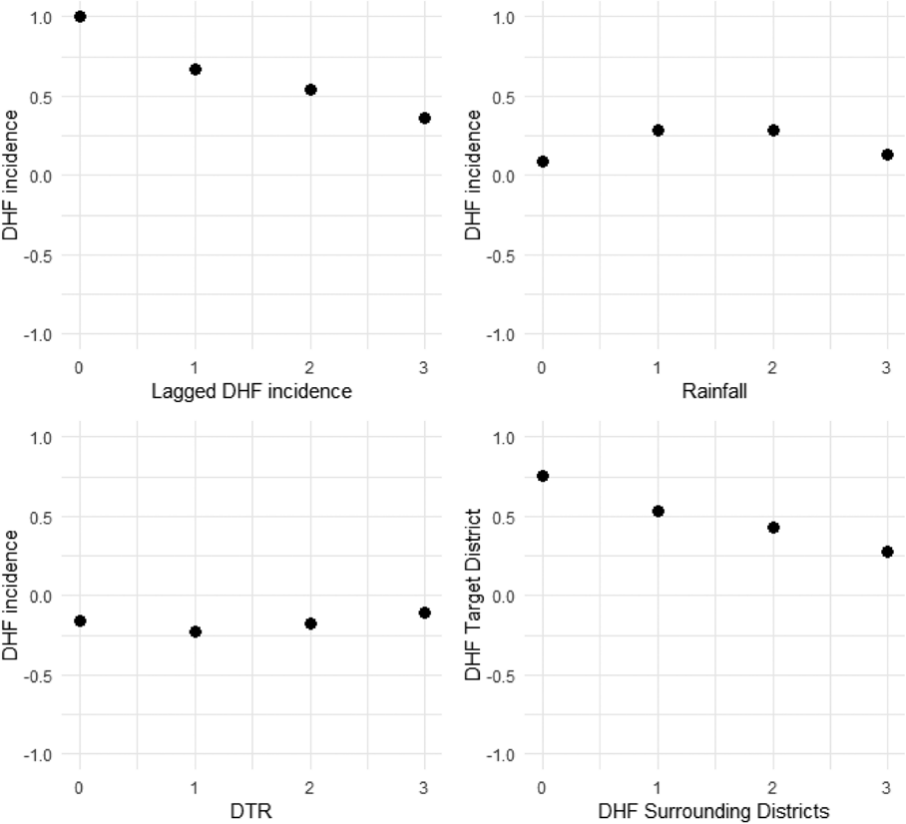
Cross-correlation between the Outcome and the Predictors

Figure 9 shows the cross-correlation cross-correlation of dengue cases with (a) dengue cases of lag of 0–3 months (b) DTR of lag of 0–3 months (c) rainfall of lag of 0–3 months and (d) the dengue cases of lag of 0–3 months from the surrounding districts. It indicates that the highest positive association between dengue incidence and lagged dengue incidences was found at lag 0 (r, 0.667), with rainfall at lag 2 (r, 0.428) and with dengue incidences from surrounding districts at lag 1 (r, 0.514). There was a negative correlation of DTR with dengue incidence at all the lags. For lag 0 (r, -0.261), lag 1 (r, -0.373), lag 2 (r, -0.309) and lag 3 (r, -0.154) was observed.

**Fig. 10:**
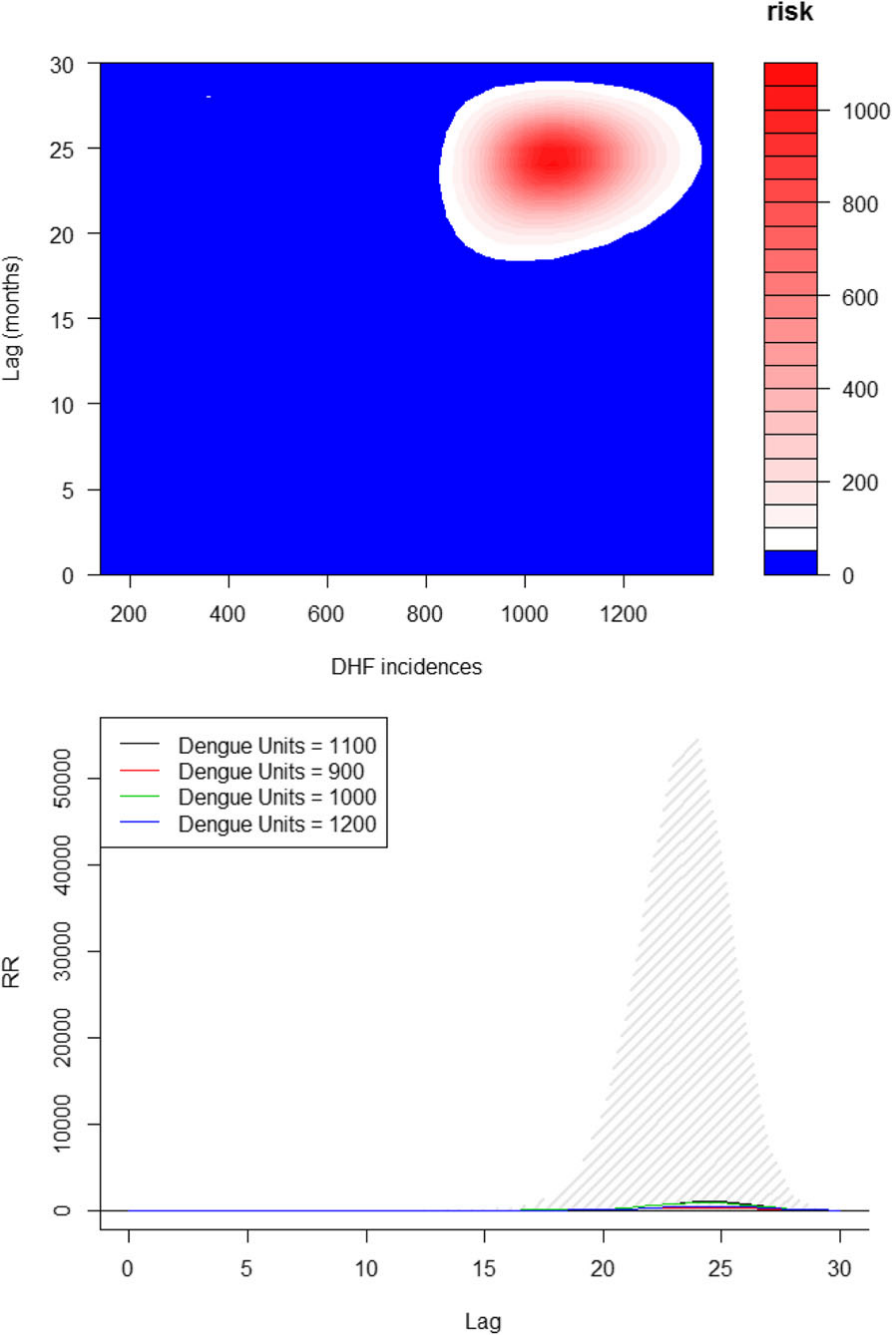
Retrospective transmission period is calculated to account for the influence of dengue incidences in each of the target districts (Fig 10) and lag-response curve for an increase in various units of dengue incidences (Fig 10)

But in order to create an early warning system (EWS), it is required to give a certain lead time. Thus, in subsequent analysis all the data that we use shall have a lag of atleast one month. The second model that we fit consists solely the data of lagged dengue incidences. For short term lags, the data from past 1−4 months was included. When the long-term lagged dengue incidence data was taken into account, the non-linear distributed lag models were used using dlnm package [73]. The influence of long-term lagged data of dengue incidences is shown using the contour plot in Fig. 10. It is observed that lagged long-term data has lower relative risks of transmission up to almost 2 years following a large outbreak in around lag 23. This suggests a negative feedback cyclic pattern. Figure 10 suggests that when an outbreak happens in a particular month then dengue risk in each of the following months will increase with a peak in next 23 months (Fig. 10). Figure 10 contains the lag-response curve for the differing number of dengue cases after an outbreak happens in a specified month. Thus based on these analyses, the optimal variables for the prediction models included dengue count at lag 1,2 for short-term lags and lag 23 for long term lag. We then combined the meteorological variables for lag greater than 1 month and dengue lag terms used in ‘Optimal Lag Surveillance’ model (model C) to determine their association with dengue incidences. This model is termed as ‘Optimal Met and Lag Surveillance Model’ (model D).

## Discussion

Since one of our hypotheses is that the significance of movement patterns of people and spatial heterogeneity of human activities on the spread of the epidemic in statistically significant. In other words, the dengue cases in a particular district are influenced by the dengue cases in there surrounding districts. To test the hypothesis, we determine how the occurrence of dengue in a target district is influenced by the occurrence of dengue in its surrounding districts. Both short-term and long-term lagged dengue cases data of the dengue incidences in surrounding districts was taken into account. For short-term lags, we considered the lagged data of past 1-4 months in which the data of lag 1 and 2 months was found to be statistically significant (p <0.05). For long-term lags, the data up to the past 30 months was used. To determine the relative risks of transmission following an outbreak in a specified month, the non-linear distributed lag models were used using dlnm package [73]. For the sake of brevity, the lag-response curve for the differing number of dengue cases is not shown here. Based on the aforementioned analyses, the optimal lag terms of dengue incidences in surrounding districts were found to be for lag 1, lag 2 and lag 12 (p ≫ 0.05). These smooth terms were combined with that of Model D and termed as ‘Optimal Representation Model’ (model E) according to Eq. 5.

5 $$  {\begin{aligned} log (C_{0,t}) \sim \alpha + \sum_{l=0}^{3} ns(\rlap{T}c_{lt}, d =3) + \sum_{l=0}^{3} ns(R_{lt}, d =3) +\sum_{l=1}^{2} ns(C_{lt}, d =3) + \\ \sum_{l=23}^{23} ns(C_{lt}, d =3) + \sum_{l=1}^{2} ns(SC_{lt}, d =3) + \sum_{l=12}^{12} ns(SC_{lt}, d =3) \end{aligned}}  $$

where *C* represents the dengue counts in the target district, T crepresents DTR (°C), *R* denotes the mean monthly rainfall (mm) and *SC* denotes the dengue counts in the surrounding districts.

**Fig. 11:**
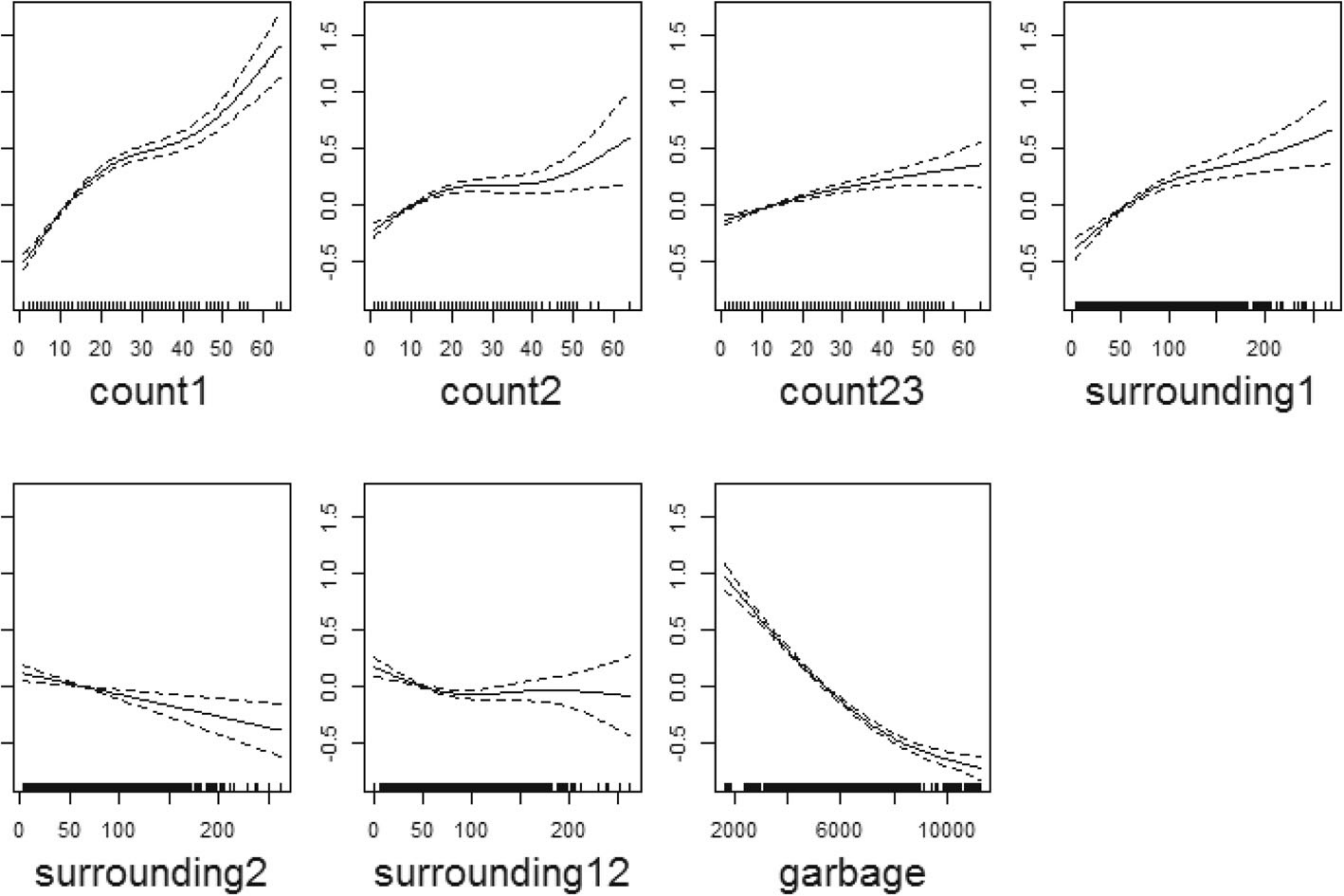
Association between past dengue count over optimal lags (lag 1,2 and 23) for each target district and lag 1,12 for surrounding district count data and garbage data with dengue outbreak. Solid lines represent relative risks (RR) of dengue cases whereas the dotted lines depict the upper and lower limits of 95% confidence intervals

**Fig. 12:**
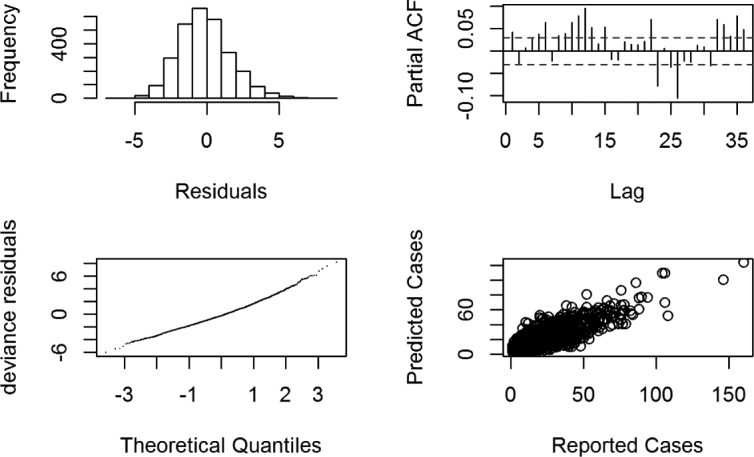
Residual Diagnosis. Top-left is the histogram of residuals; top-right is the partial ACF plot. On bottom-left is the Q-Q plot for the deviance residuals whereas the relationship between the reported and predicted cases is shown on bottom-right

Our last model (F) included waste disposal data (as a socioeconomic indicator) from each district as a predictor. The association of the smooth terms with dengue transmission is shown in Fig. 11. After the final model (F) is fit, the residuals are inferred; and their normality and residual autocorrelation are checked. Figure 12 shows that residual histograms (top-left) are symmetric and follow a unimodal distribution. The Q-Q plot of deviance residuals which are conditional on the fitted model coefficients and scale parameter (bottom-left) is close to a straight line. This suggests that the distributional assumptions of the model are satisfied. The plot of reported and predicted cases indicate linear relation (bottom-right). The partial autocorrelation function plot (top-right) does show significant autocorrelation for some long-term lags, but it was found that keeping those lagged terms increases the complexity of the model without any significant increase in prediction performance. Thus, they were chosen to be ignored in the model. These include those additional terms that do not as well provide insights in favor of our hypothesis.

The simplest Seasonal Naïve model (A) that used month of the year data for making the forecasts showed the poorest performance when compared with the other models. The quality of fit and predictive ability increases with the subsequent models as shown (Table 2).

**Fig. 13:**
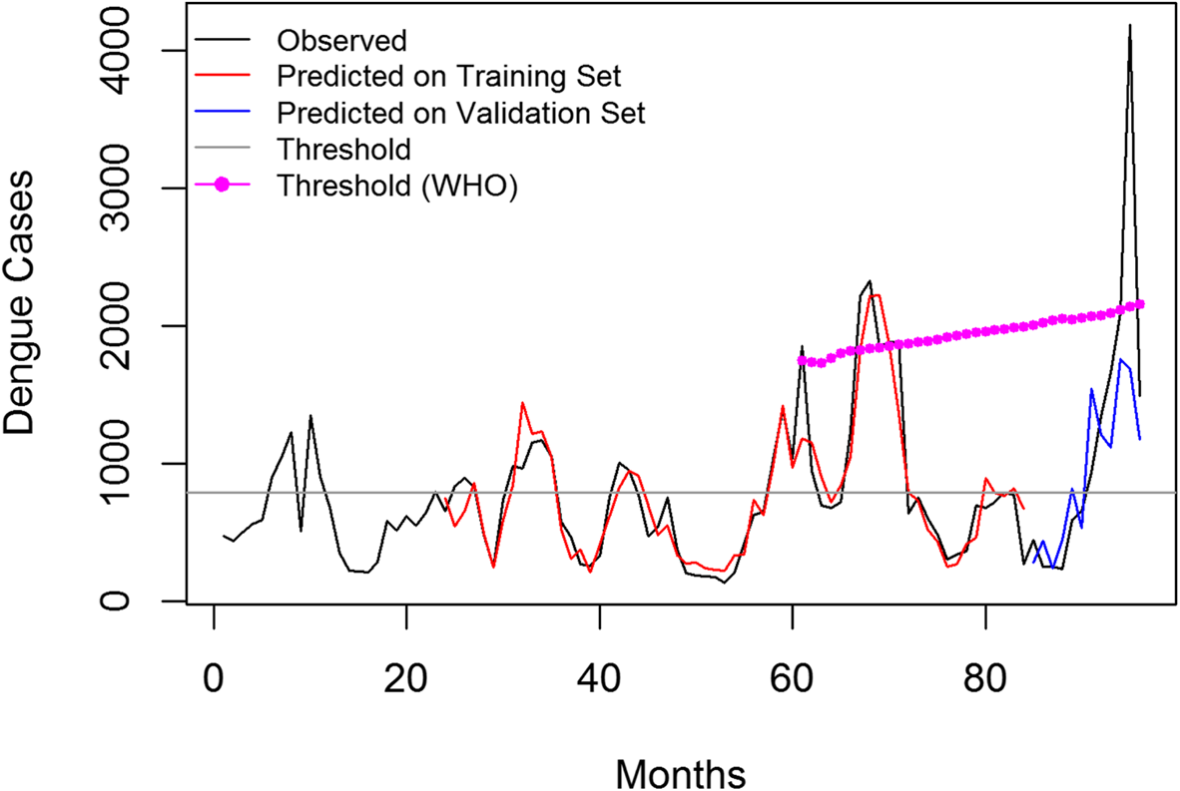
The final model (F) was trained on the data from 2008−2014 and the validation is performed on external data of year 2015

To evaluated the predictive performance of our final model (F), few external validation data sets were created for which the predictive performance is shown in Table 3. The model trained on the larger set of training data shows the better predictive performance as the influence of both, the direct and retrospective transmission could be learned in the model. For one the case in which model was trained on data from 2008−2014 and evaluation performed for year 2015, the visual analysis is shown is provided in Fig. 13.

**Table 3:** Predictive performance statistics of most optimal model evaluated on validation data sets is measured in SRMSE

Training Dataset	In-sample	Out-Sample (2014-2015)	Out-Sample (2014)	Out-Sample (2015)
2008-2013	0.41	0.62	0.35	0.60
2008-2014	0.41			0.54

The results of the discriminating ability of model (F) evaluated against the constant threshold of the epidemic (average monthly dengue cases from 2008−2015) as well WHO moving threshold is shown in Table 4. The comparison is done in terms of Sensitivity (true positive rate), Specificity (true negative rate), positive and negative predictive values (PPV and NPV respectively). In other words, Sensitivity means the proportion of observed positives that were predicted to be positive. The Specificity is the proportion of observed negatives that were predicted to be negatives. The PPV is the proportion that answers the question: ‘How likely is it that this dengue outbreak will happen given that the prediction result is positive?’ The NPV answers the question: ‘How likely is it that outbreak does not happen given that the prediction result is negative?’. Because of the higher values of the WHO threshold, the NPV is 100% as compared to that of moderate values of constant threshold. However, it should be noted that Thailand suffered a big unexpected outbreak in year 2015 for which the quality of prediction results deteriorated.

**Table 4:** The discriminating ability of the final model (F)

Prediction Accuracy	Epidemic constant threshold monthly average cases (2008−2015) = 790 cases	Epidemic moving threshold (mean of a moving window over preeding 5 years + 2 SD)
Sensitivity	87.0	100.0
Specificity	92.60	57.14
PPV	95.72	90.62
NPV	78.80	100.0

## Conclusion

In this study, the dengue incidences were predicted using a variety of data. The best model for the dengue prediction is the one that includes lagged meteorological data (rainfall, DTR), lagged dengue data of the target districts as well as their surroundings and the socioeconomic data. We proved that for the prediction of dengue outbreaks within a district, the influence of dengue incidences and socioeconomic data from the surrounding districts is statistically significant. Thus for forecasting dengue outbreaks and taking preventing measures, the epidemiologists and health authorities should consider the influence of movement patterns of people and spatial heterogeneity of human activities. The results also support previous studies that suggest temperature, precipitation, short and long term lagged incidences are related with dengue occurrence and its transmission.

A number of limitations are apparent for this study. First, the predictive model that is finally selected, could explain only 73 percent of the variation in the occurrence of dengue cases. The remaining 27 percent unexplained variation could be due to the influence of other factors. The out-of-sample predictive performance was considerably worse than that of in-sample performance. Rather than dismissing it as a case of overfitting, the case demands that we look into the facts and ‘plausible causes’ behind it. Thailand had an unexpected dengue outbreak in year 2015, the worst in last 20 years. It was the year for dengue outbreaks across Asia. Along with Thailand, other countries like Malaysia, the Philippines, Thailand, Taiwan, Vietnam and India were among the worst hit countries. According to the World Health Organization, Malaysia reported nearly 18% more cases from 2014, The Philippines reported an almost 50% rise in cases compared with 2014 and India reported double the cases as compared to the previous year. Thus, the validation dataset and training dataset did not have similar representation. Second, the study simply used monthly dengue aggregate data rather than using direct analysis of laboratory surveillance reports. The monthly dengue aggregate data constitutes only the laboratory confirmed dengue cases. But almost three-quarters of people who catch dengue have few or no symptoms. Despite being asymptomatic, these people may play a key role in the dengue transmission cycle. But our data does not represent such cases nor does it put weighted emphasis on people that suffer from multiple infections from different serotypes which puts one at greater risk for deadly severe dengue haemorrhagic fever (DHF) and dengue shock syndrome (DSS). Third, dengue severity is a key determinant of underreporting. There are several impediments of reporting dengue cases in Thailand and we need to incorporate the measure to estimate the underreporting of dengue inpatients on the district level. The results show that binary system of classifying months into outbreaks and non-outbreaks as proposed in the paper worked quite well in our evaluation. We need to add more data and collect a more diverse data. The diverse data may include House index (HI), Breteau index (BI), Container index (CI) and integrating the information from social media platforms to track the dengue incidences in real time will likely better the prediction.

Social media also has become a key component in understanding dengue. For example in 2015, popular actor Thrisadee ‘Por’ Sahawong died due to dengue infection. This case created a sense of panic which motivated people to understand the facts, symptoms, mechanism of virus transmission and the preventive measures including vaccination. We also aim to develop customized models for each individual district that includes demographic data, data from government surveys and above-mentioned additional features at a more granular level.

## Abbreviations

DENV: Dengue virus
DF: Dengue fever
DHF: Dengue haemorrhagic fever
DSS: Dengue shock syndrome
DTR: Diurnal temperature range
GAM: Generalized additive model
DLNM: Distributed lag non-linear models
RMSE: Root mean squared error
SRMSE: Standard root mean squared error
PPV: Positive predictive value
NPV: Negative predictive value
